# Effects of the Preschool-Based Family-Involving DAGIS Intervention on Family Environment: A Cluster Randomised Trial

**DOI:** 10.3390/nu12113387

**Published:** 2020-11-04

**Authors:** Carola Ray, Rejane Figueiredo, Riikka Pajulahti, Henna Vepsäläinen, Elviira Lehto, Reetta Lehto, Maijaliisa Erkkola, Eva Roos

**Affiliations:** 1 Folkhälsan Research Center, Topeliuksenkatu 20, FI-00250 Helsinki, Finland; rejane.fig@gmail.com (R.F.); riikka.pajulahti@helsinki.fi (R.P.); elviira.lehto@helsinki.fi (E.L.); reetta.lehto@folkhalsan.fi (R.L.); eva.roos@folkhalsan.fi (E.R.); 2 Department of Food and Nutrition, University of Helsinki, P.O. Box 66, FI-00014 Helsinki, Finland; henna.vepsalainen@helsinki.fi (H.V.); maijaliisa.erkkola@helsinki.fi (M.E.); 3 Clinicum, University of Helsinki, P.O. Box 63, FI-00014 Helsinki, Finland; 4 Department of Teacher Education, University of Helsinki, P.O. Box 9, FI-00014 Helsinki, Finland; 5 Department of Public Health, University of Helsinki, P.O. Box 63, FI-00014 Helsinki, Finland

**Keywords:** intervention effects, parental role modelling, family environment, availability and accessibility, energy balance-related behaviors, children, cluster randomised controlled trial, parental educational level

## Abstract

Interventions promoting young children’s healthy energy balance-related behaviours (EBRBs) should also examine changes in the family environment as this is an important determinant that may affect the effectiveness of the intervention. This study examines family environmental effects of the Increased Health and Wellbeing in Preschools (DAGIS) intervention study, and whether these effects differed when considering three parental educational level (PEL) groups. The DAGIS intervention was conducted in preschools and involving parents in Southern Finland from September 2017 to May 2018. It was designed as a randomised trial, clustered at preschool-level. Parents of 3–6-year-olds answered questionnaires recording PEL, parental role modelling for EBRBs, and the family environment measured as EBRBs availability and accessibility. Linear Mixed Models with Repeated Measures were used in order to detect intervention effects. Models included group by time interactions. When examining intervention effects separated by PEL groups, models with three-level interactions (group × time-points × PEL) were evaluated. There was an interaction effect for the availability of sugary everyday foods and drinks (*p* = 0.002). The analyses showed that the control group increased availability (*p* = 0.003), whereas in the intervention group no changes were detected (*p* = 0.150). In the analysis separated by PEL groups, changes were found only for the accessibility of sugary treats at home; the high PEL control group increased the accessibility of sugary treats (*p* = 0.022) (interaction effect: *p* = 0.027). Hence, results suggest that the DAGIS multicomponent intervention had a limited impact on determinants for children’s healthy EBRBs, and no impact was found in the low PEL group.

## 1. Introduction

Young children’s energy balance-related behaviours (EBRBs), such as physical activity (PA), screen time (ST), and food intake are highly dependent on the environment in which they spend most of their time [1,2]. Most Finnish children spend their time in mainly two settings: at home and at early childhood education and care centers, hereafter named preschools [3]. In the age 3–6-year-old age group about 78–86% attend public preschools, the proportion growing as children get older [3].

Preschool and family interventions aiming to promote healthy EBRBs among 3–6-year-olds have yielded positive effects, mostly on food intake such as increased consumption of fruit and vegetables (FV) and decreased consumption of sugar-sweetened beverages [4,5], whereas in general fewer effects have been reported on physical activity [6,7]. The interventions have focused either on one specific behaviour [8,9], or have been more complex, aiming to change several EBRBs such as reducing sugary foods and drink consumption and decreasing screen time [7,10,11]. The mixed results of the EBRB promoting interventions have been discussed, and especially the challenge of reaching parents in preschool-based family-involving interventions [9,11]. Furthermore, little knowledge exists about how to reach parents with low socio-economic position or educational level in universal interventions [12,13]. A review of universal interventions in schoolchildren concluded that, in general, interventions have not changed behaviours of children of low socio-economic position, but rather the gap between socio economic groups has enlarged [12]. In general, children with a lower parental educational level (PEL) background are more at risk of ill-health, as children with low PEL backgrounds tend to already have less healthy EBRBs at preschool-age [14,15,16,17,18].

In interventions the change mechanisms or the pathways behind the intended effects on children’s health behaviours are not always assessed, or are unknown [19,20]. To get a broader understanding of these effects, and whether they occurred or not, it is of importance also to evaluate the intervention effects on the proposed determinants for the EBRBs. This is in line with the Intervention Mapping (IM) protocol, which proposes that the key determinants need to be defined, in order to allow development of strategies to change the defined determinants [21]. Therefore, when intervening in young children’s EBRBs as the final aim, changes in parents’ behaviours, as responsible for the environment at home, should be equally assessed and evaluated. For example, in the multicomponent Toy Box preschool family-involving intervention, improvements were reported in several family determinants for children’s snacking behaviour, even though the intervention had no significant effect on children’s snacking consumption [22]. The study concluded that, even though several parenting practices were changed, a wide range of determinants should be influenced in order to influence in turn children’s snacking behaviour. An additional conclusion was that interventions could benefit from personalization, since different strategies might need to be used to reach families with different PEL backgrounds. Although these different strategies cannot be used as universal interventions, it is important for health equity to analyze whether effects differ with respect to PEL, and thus increase knowledge of intervention effects [12,20].

The Increased Health and Wellbeing in Preschools (DAGIS) intervention study used the IM protocol for planning [21]. The protocol, as with other intervention guidelines, emphasizes that a change model is needed in complex interventions [21,23]. The logic model of change includes the determinants or mediators which need to be changed, in order to reach the main aims or effects. The main aim of the DAGIS intervention was to promote 3–6-year-olds’ healthy EBRBs and self-regulation skills. This was done through a programme run in preschools which also involved families. In addition, the aim was to produce stronger effects on healthy EBRBs and self-regulation skills among children with low PEL backgrounds in order to diminish gaps in EBRBs and self-regulation skills that might exist between children with different PEL backgrounds [24]. In the DAGIS intervention study results from our needs assessment phase created the determinants of special importance for children with low PEL. The results of needs assessment were derived from mediation analyses conducted on data from the prior cross-sectional DAGIS study, results from focus groups with parents and early educators, and additionally an informal literature review [25,26,27]. The conducted focus group study and the survey were based on a socio-ecological framework developed for DAGIS [28]. The mediation analyses identified parental role modelling and home availability and accessibility of foods, outdoor PA places, and screens as determinants on which the intervention should focus. These determinants needed to change to reach a change in children’s EBRBs and self-regulation skills [24]. To get a broader knowledge of the intervention effects on children, we therefore need to examine also whether any changes occurred in defined determinants.

The aim of the study is to explore whether the DAGIS intervention had effect on children’s EBRBs environment, defined as parental role modelling, and family environment, defined as availability and accessibility at home. Further, the study aimed to explore whether the effects on parental role modelling, availability and accessibility differed when dividing parents into groups according to the PEL.

## 2. Materials and Methods

### 2.1. Aim, Design and Setting

The DAGIS intervention study was conducted in Southern Finland during the school year 2017–2018 [29] as a cluster randomised trial at the preschool level. The aim was to promote 3–6-year old children’s healthy EBRBs and self-regulation skills through activities in both preschools and at home. Simultaneously, the intervention aimed to have a stronger effect on children with low PEL backgrounds. Prospective trial registration: ISRCTN57165350 (8 January 2015)

### 2.2. Recruitment

The recruitment process for the DAGIS intervention study started at the municipality level, inviting municipalities one by one [29]. The invited municipalities had a high number of preschools and a large variety in educational and income levels among their inhabitants. Finally, the DAGIS study included preschools from two municipalities in Southern Finland, both located about 100 km from Helsinki, and both consisting of an urban city center, surrounded by a wide rural area [29]. In the municipality of Salo, all public preschools participated (*n* = 29), and in Riihimäki there were three public preschools which registered their participation as soon as we had informed the preschool headmasters about the study [29]. In total, 1702 eligible children and their families were invited to participate in the study. Consent to participate was obtained from parents of 802 children (participation rate 47%), of whom 85 were siblings. In total, the parents of 698 children filled-in the questionnaires (79 siblings), which included items related to family environment (response rate 87% of participants).

### 2.3. Ethical Issues

An ethical approval for the DAGIS intervention study was given by the Helsinki Ethical review board in Humanities and Social and Behavioral Sciences in May 2017 (22/2017; 16th of May 2017). The participating families returned their signed informed consent to preschools, prior to the start of the study

### 2.4. Data Collection

The baseline data collection started in September 2017 and lasted five weeks; the follow-up data collection was conducted in April-May 2018, also for five weeks. In each preschool the data collection took place over one week, as measuring children’s PA and screen time needed one week. Simultaneously to the measurements conducted on the children, their parents received an electronic questionnaire by email, including a personal link. Printed copies of the questionnaire were distributed and collected in closed envelopes through preschools for those parents who had so requested (*n* = 136).

### 2.5. Measurements

The original questions and the answer categories of the outcome variables and how these were formed are presented in detail in Table 1.

Parents reported their screen time and food consumption role modelling behaviour by responding to seven questions (please see Table 1). The variables based on the questions were used as mean values in analyses. The specific variable, parental role modelling for sugary treats, consisted of answers from one question, and was therefore treated as a categorical variable in the analyses. The questions used were derived from previous studies exploring parental role modelling [30,31,32]. If several children from the same family were included, the parent answered these questions separately for each child.

The availability of separate food items at home was explored in the same questionnaire (see Table 1). Three variables were formed based on the separate items describing the specific food availability at home: sugary everyday foods and drinks, sugary treats, and FV. The availability instrument has been reported in more detail elsewhere [33], and the separate items have showed moderate to good reproducibility [34]. Items were reported at family level, meaning that only one answer per family was received and independently accounted for in the analyses if there were several children participating from the same family.

Accessibility was measured for screen time behaviour, outdoor PA places, and foods (Table 1). Accessibility of screens was assessed by one question, measuring whether portable screens are kept in sight of the child. Accessibility of outdoor PA places was measured by three items describing how often parents visited outdoor PA places with their child. Child’s accessibility to food related to food visibly kept at home. The answers were grouped into accessibility of sugary everyday food and drinks, sugary treats, and FV, were then developed for the DAGIS survey study [35], and most of the items tested showed moderate to good test-retest reliability [34]. The accessibility variables were analyzed at family level, meaning that only one answer per family was used in the analyses. However, parents answered separately for each child on the three specific questions about accessibility of outdoor PA places, and the formed variable was therefore analyzed separately for each participating child.

Parents reported their highest education level by two questions; one for the respondent, and one where the responding parent reported the educational level of the partner living in the same household. The variable included three categories: the low PEL category comprising of comprehensive school, vocational school, or high school; the middle PEL category comprising of bachelor’s degree or college; and the high PEL category comprising of master’s degree or higher degree. The highest parental educational level (PEL) in the family was used in the analyses. This variable was chosen as both parents in the family influence the home environment.

### 2.6. Confounders

The analyses included confounders: the person who filled in the questionnaire (mother, father, or other), child’s gender, and the age of the child. In addition, adjustments were made for municipality. Models related to screens (role modelling and accessibility) were adjusted for the number of screen devices and paid entertainment services at home that parents reported at baseline (the sum of televisions, tablets, game consoles, DVD devices, smart phones and paid entertainment services), as the number of available screens at home can affect the intervention effects.

### 2.7. Randomization and the DAGIS Intervention Programme

The 32 participating preschools were, using an online programme, randomized into 13 intervention and 19 control preschools [29]. The DAGIS intervention lasted five months from November 2017 to April 2018. The programme was divided into five main themes aiming to impact children and their parents: strengthening self-regulation skills, enhancing PA, promoting FV consumption, reducing excessive screen time, and restricting the consumption of sugary everyday food and drinks and sugary treats. The materials have been described in detail elsewhere [24]. Basically, the materials for parents focused on getting parents to reflect on their own EBRBs as role models for children’s EBRBs, giving hints on how to change the family environment in order to promote healthy EBRBs, and enhancing knowledge about healthy EBRBs. The programme also aimed to increase social support between parents by offering a mobile application based on geographic information system (GIS). The application encouraged parents to mark out PA enhancing outdoor places for children in the municipality, and thereafter share it with other parents. All produced materials were easy to read and the texts were short, including sentences which were clear and concise. In addition, many of the methods used, such as doing things together with the child or families supporting each other, have worked well among low PEL families [36,37].

### 2.8. Statistical Analyses

Linear Mixed Models with Repeated Measures were used in order to detect intervention effects, and the comparisons between baseline and follow-up in the intervention and the control group were presented. In models with quantitative outcomes the normal distribution of the outcomes was checked for the quantitative variables. For the two categorical variables in the study (parental role modelling of sugary treats and accessibility of screen devices for the child), a similar mixed model with repeated measures specific for ordinal variables was used. In all models, adjustments were made for who filled in the study (mother, father, or other), PEL and municipality. In models with variables derived at child level (i.e, when parents answered separately for all of their children in the study), models were also adjusted for gender and age of the child. Preschool and family were used as random effects. For dependent variables related to screens (parental role modelling and accessibility of screens), the models were also adjusted for number of devices and screen services at home (televisions, tablets, game consoles, DVD devices, smart phones and paid entertainment services). The models included an interaction between the group (control/intervention) and the time-points (baseline/follow-up). In addition, to be able to detect changes from baseline to follow-up by PEL, models with three-level interactions were carried out: groups (control/intervention), time-points (baseline/follow-up), and PEL groups. In the results, in order to underline the intervention effect separated by groups and PEL groups, we presented the estimated change in the outcome variable between baseline and follow-up. The calculated power varied from 74.1% to 92.4%. 

Missing values in the models were replaced using multivariate imputation by chained equations (MICE), considering the missing as completely at random [38]. The following independent variables had missing values: PEL, who filled in the questionnaire, and the age of the child (in models where parents had answered for each participating child separately) (Supplementary Table S1). In the Mixed Models, the information for all participants who had data on at least one of the measured outcomes, at baseline or at follow-up, was considered. All analyses were based on the intention-to-treat principle, which meant that all participants were included in the analysis on the basis of which group they were randomized into.

IBM SPSS Statistics for Windows, Version 25.0 was used for descriptive analyses (IBM Corp, Armonk, NY, USA). Mixed models, mixed models for ordinal variables and multiple imputation analysis were conducted in R version 3.4.3 using lme4 [39], Ordinal [40] and MICE packages [38], respectively. For all analyses a 5% statistical significance level was adopted.

## 3. Results

### 3.1. Participants

Usually, it was the mother of the child who answered the questionnaire (80%), and most of the families were two parent families (71%) (Table 2). The distribution of families by PEL was as follows: about 22% of the families were classified in the highest educational level, 43% were classified as middle educated, and 31% were classified as low PEL. The mean age of the respondents was about 35.9 years (SD 5.4), and the age of the child 5.2 (SD 1.0) years. About 47% of the children were girls and 53% boys.

The number of respondents may vary, as some items were answered at family level, whereas others were answered by the parent separately for each child. The 79 siblings participating in the study therefore increased the number of responses for certain items: parental role modellings, keeping screens in sight of a child, and visits to outdoor PA places.

### 3.2. Descriptives of Role Modelling, Accessibility, and Availability

Table 3 shows the descriptors of outcomes at baseline and follow-up for each role model, availability and accessibility outcome. Supplementary Table S2a,b presents descriptors of the family environment separated by PEL groups (Table S2a for quantitative variables and Table S2b for categorical variables). In general, parents used screens for about 75 min/day in sight of the child. Parents reported that they consumed fruit and vegetables slightly more than 6 times/week, when the child was around.

### 3.3. Intervention Effects on Role Modelling, Availability, and Accessibility in Control and Intervention Groups and Changes in Control and Intervention Groups

Table 4 shows the results of intervention and changes in role modelling, availability, and accessibility from baseline to follow-up in the control and intervention groups. In the control group the availability of sugary everyday foods and drinks increased from baseline to follow-up (*p* = 0.003), whereas no change was detected in the intervention group (*p* = 0.150). The interaction between group and time was statistically significant (*p* = 0.002), showing an intervention effect. No other statistically significant results were detected for the interaction term group over time. However statistically significant changes were detected in several behaviours in both groups. Both the control and the intervention group decreased the number of visits to outdoor PA places (*p* < 0.001 and *p* < 0.001). Similarly, both control and intervention group decreased the accessibility of sugary everyday foods and drinks (*p* = 0.006 and *p* = 0.002). In the intervention group, the estimate of the decrease was -0.14, whereas the estimate in the control group was −0.11. A borderline significant result (*p* = 0.052) was detected in the intervention group in increasing the availability of fruit and vegetables at home. No other significant results were detected.

### 3.4. Intervention Effects on Role Modelling, Availability, and Accessibility in Control and Intervention Groups, and Separated by PEL and Changes in the Control and Intervention PEL Groups

Table 5 shows the results of intervention and changes in role modelling, availability, and accessibility from baseline to follow-up in control and intervention groups separated by PEL. The interaction over group, time points and PEL showed statistical significance for accessibility to sugary sweets (*p* = 0.027) (see Table 5). An increase in accessibility of sugary treats was observed in the high PEL control group (*p* = 0.022), whereas in the corresponding intervention group no change was detected (*p* = 0.393). There also seemed to be a decrease in the accessibility of sugary treats in the low PEL intervention group, but the change was not statistically significant (*p* = 0.098). The other interactions (groups over time points over PEL) were not statistically significant.

However, some changes occurred in the PEL groups. In the high PEL control group, there was an increase in parental role modelling for consumption of sugary everyday food and drinks consumption by an estimate of 0.60 times per week (*p* = 0.029). No change was detected in the high PEL intervention group (*p* = 0.211).

Both the low and the middle PEL control groups increased the availability of sugary everyday foods and drinks (*p* = 0.026 and *p* = 0.037), even though the estimates were low. No similar results were observed in low and middle PEL intervention groups (*p* = 0.663 and *p* = 0.790). The high PEL intervention group decreased the availability of sugary everyday foods and drinks (*p* = 0.028), whereas no change was detected in the high PEL control group (*p* = 0.381).

The accessibility to outdoor PA places decreased in the low and middle PEL control groups (*p* = 0.018 and *p* = 0.023, respectively). Similarly, a decrease was found in the middle PEL intervention group (*p* = 0.002). A decrease was not detected in the low PEL intervention group, but the *p* value was borderline significant (*p* = 0.056). The interaction was not statistically significant (*p* = 0.558). The low PEL control group decreased the accessibility of sugary everyday foods and drinks (*p* = 0.019), whereas in the low PEL intervention group no changes were detected (*p* = 0.174). However, a decrease in accessibility of sugary everyday foods and drinks was observed in the middle PEL intervention group (*p* = 0.001), whereas no change was detected in the middle PEL control group (*p* = 0.305). The interaction term was not statistically significant (*p* = 0.124).

## 4. Discussion

The first aim of the study was to explore whether the DAGIS intervention had an impact on parents as role models for children’s EBRBs, and whether the family environment, assessed as availability and accessibility of healthy EBRBs changed. The second aim was to explore whether the intervention impact differed by parental education in the family.

Overall, intervention effects were low on parental role modelling and the family environment. The two main intervention results were related to the family food environment, as the availability and accessibility of foods changed. Firstly, in the intervention group, the availability of sugary everyday food and drinks at home did not change: meanwhile an unfavorable change, an increase, occurred in the control group. Secondly, an interaction effect was detected in the PEL-stratified analyses: the accessibility of sugary treats increased in the high control PEL group, whereas no changes occurred in the high intervention PEL group. Even though there were no intervention effects, some changes related to availability or accessibility of foods in the separate PEL groups were pointed out, such as the increase in availability of sugary everyday food and drinks in the low and the middle PEL control groups. However, the availability of sugary everyday foods and drinks decreased in the high PEL intervention group. In addition, the accessibility of sugary everyday foods and drinks decreased in the middle PEL intervention group, as in the low PEL control group.

The results can be interpreted as the intervention impacting on the main themes in the DAGIS logic model of change, i.e., the availability of sugary everyday foods and drinks, albeit only modestly (see [24]). There was no change in the intervention group, whereas the control group increased the availability of these food and drinks. In the separate PEL groups some changes occurred; there was a decrease in the high PEL intervention group, whereas the low and middle PEL control groups increased availability, even though no interaction was detected. Still, the results conflict with the second aim of the intervention, namely to have higher effects in the low PEL group [24]. Our results showed, similar to those of other studies [12,13], the challenges of intervening in families with low PEL. At this point we can only speculate on what can have been the reasons for not producing the aimed effects in low PEL groups. One reason could be that the activities in the program were not implemented in low PEL families. In addition, even though there was a focus on developing a programme which would appeal to low PEL families, these families might not have felt a need for change, or were not motivated to change, or did not have the self-efficacy for change. Therefore, a comprehensive process evaluation including the intervention implementation degree by PEL groups is needed, in order to obtain a deeper understanding of the impact of the intervention by PEL group.

Parental role modelling for eating habits, and the availability of foods have, in many published studies [33,41,42,43] and reviews [44,45,46], shown to be strong determinants for children’s healthy or unhealthy food consumption. The availability of unhealthy foods at home has also been associated with unhealthy diet among children, regardless of the availability of healthy foods at home [33,47,48]. In the control group, the availability of sugary everyday foods and drinks increased and, in addition, in the high PEL control group parental role modelling for consumption of sugary everyday foods and drinks increased. This might affect children’s food consumption negatively. The expected mechanism in the DAGIS logic model of change was that, prior to a change in children’s EBRBs, the primary outcomes, parental role modelling, home availability and accessibility, need to change [24]. The presumed pathways to children’s food consumption in the DAGIS intervention study should therefore be tested further by mediation analyses, as previously discussed [19,20], even though the main effects on children’s food consumption in the DAGIS study showed no statistically significant results. Still, the study of intervention effects on children’s EBRBs in the DAGIS study showed a tendency towards increasing fruit and vegetable consumption among children in the intervention group [29].

There were no intervention effects on parents’ behaviours regarding screen time, such as parental role modelling of screen time or the accessibility of screens. Reducing excessive screen time among children was one of the main objectives of the intervention [24]. In the DAGIS cross-sectional study, parent’s own screen time behaviour was one of the most important mediators between PEL and children’s screen time [17,35]. In the intervention, effort was placed on reaching families, with a message to reduce children’s excessive screen time. This was done by practical actions; for instance, each family obtained feedback about child’s screen time at the baseline, a home letter with motivational and reflective contents about how to reduce screen time, emails for parents to reflect on themselves as role models and screen users, bingos enhancing PA activities, etc. One explanation why parental role modelling for screen time did not change could be that even though the materials emphasized that children learn by watching, parents might have seen the programme as aiming to change the child’s behaviour, and therefore did not feel the need to change their own behaviour. Another explanation could be that parents mostly used screens for their work tasks, even though they were asked about leisure time, and as a consequence they were not able to reduce their screen use in the presence of the child. However, the parents’ questionnaire included several other items related to screen time, such as the perceived need for change, norms, restrictive practices, self-efficacy, etc. Further studies should also explore these topics, in order to better understand the reason why the intervention had no effects on screen time.

The importance of changes in parental behaviour was highlighted in a previous study which aimed to increase children’s FV intake [41]. It concluded that in order to increase children’s FV consumption, studies should focus both on parents providing their children with FV and on improving parents’ own FV intake. We found no significant intervention effects on parental role modelling or availability/accessibility of FV. The intervention might not have reached or engaged parents, or parents might not have been interested in that specific intervention component, or felt no need for change [9,46]. The availability and accessibility of FV at home was already at baseline at quite a high level. In addition, even though both food availability and the accessibility were explained in the materials, parents might not have understood what it means in practice that FV are easily accessible, and sugary everyday foods, drinks and treats less easily accessible.

Previous studies have shown that the availability of unhealthy foods is associated with children’s unhealthy diet [33,47,48]. Our results showed that an intervention may possibly prevent an increase in the availability of sugary everyday foods and drinks at home. Therefore, further interventions should focus on developing strategies to impact food availability.

The DAGIS intervention study has several strengths, such as the development work being based on the IM framework [21]. Firstly, a logic model of change was developed for the intervention, showing the expected mechanisms behind the changes in children’s EBRBs. The intended changes in parents behaviours followed the logic model of change reflecting behavioral theories [24]. Secondly, a strength was that the model of change was developed based on a comprehensive needs assessment phase, that, among others, included mediation analyses [24,28,35]. The needs assessment has been seen as a highly important phase, enabling the planning of systematic interventions and also facilitating further evaluation [21,49]. Thirdly, a major part of the instruments assessing the outcome variables has been used in other studies and interventions, and have shown good validity and reliability [30,31,33,34]. Fourth, the participation rate was fairly high (47%), and the sample included families with diverse PEL backgrounds. In addition, multiple imputation was used in analyses for some variables. Using multiple imputation has the advantages of reducing bias, increasing precision, and getting more robust statistical results [50].

A limitation of the study is related to the development of the content in the intervention programme. The multicomponent intervention aimed to change several determinants (parental factors) in order to change children’s EBRBs. There can be a risk that parents had too many messages split into several topics, and therefore had difficulties in understanding the main objectives of the intervention. Therefore, more in-depth evaluations about the processes in the intervention are needed, in order to get a better understanding of the intervention effects. Another limitation might be that parental involvement in the intervention was not mandatory. A recently published review pointed out the importance of having parents intensively participating in interventions when promoting children’s EBRBs in preschools [4]. The intervention lasted 23 weeks, which might have been too short for the ambitious aim of detecting measurable changes at parent’s level, or at child’s level. In addition, as baseline and follow-up measurements took place during different seasons, this might have affected results. In April-May evenings are less dark than in October, which might lead to parents letting their half-year older children play outside alone, and therefore reporting less outdoor activities with their child. Sample size calculations were made for the DAGIS study, but they were based on the aim of detecting changes in children’s screen time and sugary everyday foods and drinks consumption [24]. The number of the participants needed for meeting these aims might not have been high enough, thus limiting the possibilities of detecting changes in family environment. Furthermore, the results should be interpreted with caution since the magnitude of differences were low, and due to that the multiple testing was not properly corrected. In addition, the baseline average for some of the measured behaviours in this study, such as the children’s behaviours, were already at a good level [29], which meant that there was not much room for improvement. In reporting food consumption an under- or overestimation is very common [51,52], and this could have been the case for the used food-related variables in this study.

## 5. Conclusions

This study showed that the DAGIS intervention had some effects on food availability and accessibility of foods at home, even though these effects were small. In the DAGIS logic model of change, availability and accessibility were important determinants which need to be changed in order to change children’s EBRBs. The DAGIS intervention did not detect any noteworthy effects on parental role modelling. The second aim of having stronger intervention effects among low PEL groups was not met. Hence, results suggest that the five months DAGIS multicomponent intervention had limited impact on some of the family determinants for children’s healthy EBRBs. However, no impact in the low PEL group was found. The next step will be to plan for future investigation of the intervention dose, in order to obtain a deeper understanding of the impact of the intervention by PEL groups.

## Figures and Tables

**Table 1:** Questions on parental role modelling, availability, and accessibility, answer categories, and the formation of the variables used in analyses.

Family Environment	Question	Behaviour/Food Items	Answer Categories	Formation of Variables	Variable in Analyses
Parental role modelling	About how many hours a weekday/weekend day do you usually use electronic devices during leisure time when your child is around?	Screen time	not at all, less than 30 min, between 30 min and 1 h, 1–2 h, 3–4 h, and 5 h or more	Conversion into 0, 15, 45, 90, 210, and 360 min for weekday and weekend separately	A weekly mean (5 × weekday + 2 × weekend/7)
	During the past week how often did you consume, when your child was around?	Sugary foods/drinks ^a^	not once, 1–2 times/week, 3–4 times/week, 5–6 times/week, daily, and more than once a day	Conversion into 0, 1.5, 3.5, 5.5, 7, and 10.5. The two items were summed up	A mean value
		Sugary treats ^b^	not once, 1–2 times/week, 3–4 times/week, 5–6 times/week, daily, and more than once a day	Asked as one item. Conversion into 0, 1.5, 3.5, 5.5, 7, and 10.5	Categorical
		Fruit/vegetables	not once, 1–2 times/week, 3–4 times/week, 5–6 times/week, daily, and more than once a day	Conversion into 0, 1.5, 3.5, 5.5, 7, and 10.5 The two items were summed up	A mean value
Availability	How often have you had the following foods at home during last month?	Sugary foods/drinks ^a^	never, rarely, sometimes, often, and always	Each item; min 1, max 5, nine items summed up	A mean value
		Sugary treats ^b^	never, rarely, sometimes, often, and always	Each item; min 1, max 5, seven items summed up	A mean value
		Fruit/vegetable ^c^	never, rarely, sometimes, often, and always	Each item; min 1, max 5, four items summed up	A mean value
Accessibility	In our family portable screens (e.g., tablets, phones) are kept in sight of the child		strongly agree, somewhat agree, neither disagree or agree, somewhat disagree, and strongly disagree	One item; min 1: strongly disagree, max 5: strongly agree	Categorical
	How often during the last month has your child visited the following places with at least one adult in the family?	1. Nature/forest 2. Park, playground 3. Own yard	not once, 1–3 times a month, 1–2 times a week, 3–6 times week, and daily	Three items; min 1 point, max 5 points summed up	A mean value
	If you had the foods and drinks at home did you keep them in sight of the child?	1. Sugar-sweetened cereals or muesli (more than 10g/100g sugar), 2. Sugary juices, 3. Soft drinks	no or yes	Three items; no = 0, yes = 1, summed up	A mean value
		1. Cookies etc., 2. Sweet pastries, 3. Chocolate and sweets	no or yes	Three items; no = 0, yes = 1, summed up	A mean value
		1. Fresh fruits, 2. Fresh vegetables	no or yes	Two items; no = 0, yes = 1, summed up	A mean value

^a^ 1. Sugary everyday foods: Yoghurts, quarks etc., plant-based with added sugar, puddings, and sugar-sweetened cereals or muesli (more than 10g/100g sugar), 2. Sugary drinks: 100% juices, juices with added sugar, soft drinks, and berry/fruit soups with added sugar. ^b^ Cookies, snack bars (e.g., muesli bars), cakes, muffins, buns and sweet pastry, chocolate and sweets, and ice cream. ^c^ Fruits and/or berries (fresh and frozen), vegetables (fresh and frozen).

**Table 2:** Characteristics of the participants at baseline.

		*n*	Mean ± SD
Age of the respondent, years		611	35.9 ± 5.4
		N	%
Highest educational level in the family ^a^	Low	225	31%
	Middle	312	43%
	High	159	22%
Person who answered the questionnaire	Mother	567	80%
	Father	57	8%
	Other guardian	4	1%
Family type (the child lives with...)	both parents	506	71%
	only with his/her mother	56	8%
	only with his/her father	1	0.1%
	his/her mother and her new partner	29	4%
	half time with me and half time with the other parent	24	3%
	other adults	3	0.4%
Municipality	Salo	590	83%
	Riihimäki	122	17%
Child’s gender	Girl	375	47%
	Boy	426	53%
		N	mean ± SD *
Child’s age, years		721	5.2 ± 1.0

* SD: Standard deviation. ^a^ low educational level (comprehensive school, vocational school, or high school), middle (bachelor’s degree or college), high (master’s degree or licentiate/doctor).

**Table 3:** Descriptors of outcomes-family environment.

		Baseline				Follow-Up			
		Control		Intervention		Control		Intervention	
		*n*	Mean ± SD	*n*	Mean ± SD	*n*	Mean ± SD	*n*	Mean ± SD
Parental role modelling in presence of child	Screen time (min/day)	383	77.48 ± 53.41	314	76.56 ± 45.47	323	74.69 ± 58.63	255	72.51 ± 42.28
	Parental consumption of sugary everyday food and drinks * (times/week)	384	2.37 ± 2.33	313	2.22 ± 2.40	324	2.67 ± 2.84	256	2.33 ± 2.47
	Parental consumption of sugary treats **	***n***	**%**	***n***	**%**	***n***	**%**	***n***	**%**
	not at all	101	26.3%	86	27.5%	76	23.5%	61	23.9%
	1–2 times/week	222	57.8%	192	61.3%	181	55.9%	147	57.6%
	3–4 times/week	51	13.3%	25	8.0%	49	15.1%	36	14.1%
	5–6 times/week	7	1.8%	5	1.6%	12	3.7%	2	0.8%
	once everyday	1	0.3%	4	1.3%	6	1.9%	9	3.5%
	more than once a day	2	0.5%	1	0.3%	0	0%	0	0%
	Fruit and vegetables consumption (times/week)	384	6.30 ± 2.76	314	6.10 ± 2.70	324	6.32 ± 2.68	256	6.10 ± 2.78
Availability	Sugary everyday food and drinks at home * (1–5)	341	2.55 ± 0.65	279	2.59 ± 0.61	291	2.64 ± 0.65	228	2.51 ± 0.59
	Sugary treats at home ** (1–5)	341	2.84 ± 0.70	279	2.83 ± 0.63	291	2.87 ± 0.65	228	2.83 ± 0.64
	Fruit and vegetables at home (1–5)	341	4.11 ± 0.62	279	4.02 ± 0.67	291	4.17 ± 0.63	228	4.11 ± 0.67
Accessibility	Visits to outdoor PA places (1–5)	384	3.16 ± 0.65	314	3.16 ± 0.66	324	3.04 ± 0.72	255	3.01 ± 0.70
	Sugary everyday food and drinks in-sight of the child * (1–2)	326	1.02 ± 0.56	265	1.07 ± 0.55	275	1.08 ± 0.49	223	1.02 ± 0.57
	Sugary treats in-sight of the child ** (1–2)	318	1.21 ± 0.46	249	1.20 ± 0.44	272	1.24 ± 0.46	215	1.21 ± 0.46
	Fruit and vegetables in-sight of the child (1–2)	318	1.86 ± 0.27	265	1.85 ± 0.28	278	1.83 ± 0.28	217	1.87 ± 0.26
	Parental consumption of sugary treats **	*n*	%	*n*	%	*n*	%	*n*	%
	strongly agree	158	46.5%	128	45.9%	136	46.9%	93	41.0%
	somewhat agree	118	34.7%	107	38.4%	102	35.2%	91	40.1%
	neither disagree or agree	20	5.9%	17	6.1%	18	6.2%	17	7.5%
	somewhat disagree	25	7.4%	13	4.7%	22	7.6%	14	6.2%
	strongly disagree	19	5.6%	14	5.0%	12	4.1%	12	5.3%

SD: Standard deviation. * Sugary everyday food and drinks: yoghurts, quarks etc., plant-based products with added sugar, puddings, and sugar-sweetened cereals or muesli (more than 10 g/100 g sugar), 100% juices, sugar-sweetened juices, soft drinks, and berry/fruit soups with added sugar ** Sugary treats: cookies, snack bars (e.g., muesli bars), cakes, muffins, buns and sweet pastry, chocolate and sweets, and ice cream.

**Table 4:** Comparison of parental role modeling, availability and accessibility between baseline and follow-up in control and intervention groups (*n* = 625–722).

		Linear Mixed Models with Repeated Measures						*p*-Value Interaction (Group × Time-Points)
		Change between Follow-Up and Baseline in Control Group			Change between Follow-Up and Baseline in Intervention Group			
Family Environment		Adjusted Change Mean ^†^	95% C.I. for Adjusted Change Mean	*p*-Value	Adjusted Change Mean ^†^	95% C.I. for Adjusted Change Mean	*p*-Value	
Parental role modelling	Screen time (min/day) ^a,c^	−2.40	(−7.19; 2.40)	0.327	−2.65	(−7.95; 2.66)	0.327	0.945
	Parental consumption of sugary everyday food and drinks (times/week) ^a^	0.22	(−0.06; 0.50)	0.118	0.17	(−0.14; 0.47)	0.288	0.797
	Parental consumption of sugary treats (times/week) ^a^	0.27	(−0.04; 0.58)	0.315	0.44	(0.09; 0.797)	0.0.67	0.488
	Parental fruit and vegetables consumption (times/week) ^a^	−0.02	(−0.31; 0.26)	0.863	0.04	(−0.27; 0.35)	0.803	0.763
Availability	Sugary everyday food and drinks at home (1–5) ^b^	0.09	(0.03; 0.15)	0.003	−0.05	(−0.11; 0.02)	0.150	0.002
	Sugary treats at home (1–5) ^b^	0.02	(−0.05; 0.09)	0.657	−0.01	(−0.08; 0.07)	0.888	0.687
	Fruit and vegetables at home (1–5) ^b^	0.04	(−0.02; 0.11)	0.204	0.07	(0.00; 0.15)	0.052	0.553
Accessibility	Portable screens kept in-sight of the child ^b,c^	0.02	(−0.28; 0.32)	0.999	0.24	(−0.09; 0.57)	0.476	0.333
	Visits to outdoor PA places (1–5) ^a^	−0.12	(−0.19; −0.06)	<0.001	−0.12	(−0.20; −0.05)	<0.001	0.941
	Sugary everyday food and drinks (1–2) ^b^	−0.11	(−0.19; −0.03)	0.006	−0.14	(−0.23; −0.05)	0.002	0.616
	Sugary treats (1–2) ^b^	0.06	(−0.01; 0.13)	0.094	−0.04	(−0.11; 0.04)	0.309	0.061
	Fruit and vegetables (1–2) ^b^	−0.03	(−0.06; 0.01)	0.119	0.02	(−0.02; 0.06)	0.357	0.084

95% C.I. = 95% confidence intervals. PA physical activity. ^†^ estimated change between follow-up and baseline for quantitative variables and estimated parameter for the change effect for the categorical variables “Parental consumption of sugary treats” and “Portable screens kept in-sight of the child”. ^a^ models adjusted for parental educational level, municipality, child’s gender, child’s age, and who filled in the questionnaire. ^b^ models adjusted for parental educational level, municipality, and who filled in the questionnaire. ^c^ models also adjusted for the number of screens and screen entertainment services at home.

**Table 5:** Comparison of parental role modeling, availability and accessibility between baseline and follow-up in intervention and control groups separated by parental educational level (PEL) (*n* = 625–722).

			Linear Mixed Models with Repeated Measures						*p*-Value Interaction (Group × Time-Points × PEL)
	Family Environment	PEL	Comparison between Follow-Up and Baseline in Control Group			Comparison between Follow-Up and Baseline in Intervention Group			
			Adjusted Change Mean ^†^	95% C.I. for Adjusted Change Mean	*p*-Value	Adjusted Change Mean ^†^	95% C.I. for Adjusted Change Mean	*p*-Value	
Parental role modelling	Screen time (min/day) ^a,c^	low	−4.46	(−13.79; 4.87)	0.349	−5.97	(−15.25; 3.30)	0.206	0.972
		middle	−0.09	(−7.10; 6.93)	0.981	0.38	(−7.35; 8.12)	0.923	
		high	−4.19	(−13.51; 5.13)	0.378	−4.93	(−16.84; 6.97)	0.416	
	Parental consumption of sugary everyday food and drinks (times/week) ^a^	low	0.12	(−0.41; 0.66)	0.654	−0.07	(−0.60; 0.47)	0.801	0.715
		middle	0.05	(−0.35; 0.46)	0.807	0.22	(−0.23; 0.67)	0.337	
		high	0.60	(0.06; 1.13)	0.029	0.44	(−0.25; 1.13)	0.211	
	Parental consumption of sugary treats (times/week) ^a^	low	0.16	(−0.42; 0.74)	>0.999	0.06	(−0.53; 0.65)	>0.999	0.817
		middle	0.23	(−0.21; 0.67)	0.998	0.60	(0.11; 1.09)	0.395	
		high	0.46	(−0.12; 1.05)	0.927	0.40	(−0.36; 1.15)	0.997	
	Parental fruit and vegetables consumption (times/week) ^a^	low	−0.21	(−0.75; 0.33)	0.451	0.32	(−0.22; 0.86)	0.246	0.061
		middle	0.02	(−0.39; 0.43)	0.938	−0.44	(−0.89; 0.01)	0.057	
		high	0.09	(−0.45; 0.63)	0.739	0.69	(−0.004; 1.39)	0.051	
Availability	Sugary everyday foods and drinks at home (1–5) ^b^	low	0.13	(0.02; 0.24)	0.026	−0.02	(−0.14; 0.09)	0.663	0.638
		middle	0.09	(0.01; 0.17)	0.037	−0.01	(−0.10; 0.08)	0.790	
		high	0.05	(−0.06; 0.16)	0.381	−0.16	(−0.30; −0.02)	0.028	
	Sugary treats at home (1–5) ^b^	low	0.03	(−0.11; 0.17)	0.671	−0.04	(−0.18; 0.09)	0.538	0.657
		middle	−0.003	(−0.11; 0.10)	0.950	−0.02	(−0.14; 0.09)	0.673	
		high	0.04	(−0.10; 0.17)	0.589	0.10	(−0.07; 0.27)	0.255	
	Fruit and vegetables at home (1–5) ^b^	low	0.01	(−0.12; 0.14)	0.907	0.08	(−0.05; 0.21)	0.211	0.738
		middle	0.02	(−0.08; 0.12)	0.702	0.06	(−0.05; 0.16)	0.296	
		high	0.12	(−0.01; 0.25)	0.064	0.09	(−0.08; 0.25)	0.301	
Accessibility	Portable screens kept in-sight of the child ^b,c^	low	−0.12	(−0.69; 0.44)	>0.999	0.80	(0.23; 1.37)	0.197	0.134
		middle	0.30	(−0.15; 0.75)	0.980	0.05	(−0.43; 0.53)	>0.999	
		high	−0.26	(−0.83; 0.32)	0.999	−0.25	(−1.01; 0.51)	>0.999	
	Visits to outdoor PA places (1–5) ^a^	low	−0.16	(−0.28; −0.03)	0.013	−0.12	(−0.25; 0.01)	0.056	0.558
		middle	−0.11	(−0.21; −0.02)	0.018	−0.16	(−0.27; −0.06)	0.002	
		high	−0.10	(−0.23; 0.02)	0.106	−0.03	(−0.19; 0.13)	0.680	
	Sugary everyday food and drinks (1–2) ^b^	low	−0.19	(−0.34; −0.03)	0.019	−0.11	(−0.27; 0.05)	0.174	0.124
		middle	−0.06	(−0.18; 0.06)	0.305	−0.22	(−0.35; −0.09)	0.001	
		high	−0.13	(−0.28; 0.02)	0.100	−0.02	(−0.22; 0.18)	0.840	
	Sugary treats(1–2) ^b^	low	0.11	(−0.02; 0.24)	0.108	−0.11	(−0.24; 0.02)	0.098	0.027
		middle	−0.03	(−0.13; 0.07)	0.552	0.03	(−0.08; 0.13)	0.624	
		high	0.15	(0.02; 0.28)	0.022	−0.07	(−0.23; 0.09)	0.393	
	Fruit and vegetables(1–2) ^b^	low	0.04	(−0.02; 0.11)	0.205	0.03	(−0.03; 0.10)	0.327	0.444
		middle	−0.05	(−0.10; 0.00)	0.065	0.01	(−0.05; 0.07)	0.760	
		high	−0.06	(−0.13; 0.01)	0.074	0.01	(−0.07; 0.10)	0.748	

95% C.I. = 95% confidence intervals. PA physical activity. ^†^ estimated change between follow-up and baseline for quantitative variables, and estimated parameter for the change effect for the categorical variables “Parental consumption of sugary treats” and “Portable screens kept in-sight of the child”. ^a^ models adjusted for municipality, child’s gender, child’s age, and who filled in the questionnaire. ^b^ models adjusted for municipality, and who filled in the questionnaire. ^c^ models also adjusted for the number of screens and screen entertainment services at home.

## Supplementary Materials

The following are available online at https://www.mdpi.com/2072-6643/12/11/3387/s1, Supplementary Table S1: Number of children and missing values, Table S2a: Descriptive of outcomes-family environment by parental educational level (quantitative variables), Table S2b: Descriptive of outcomes-family environment by parental educational level (categorical variables).
