# Supplementary material for: Effects of the Preschool-Based Family-Involving DAGIS Intervention on Family Environment: A Cluster Randomised Trial
Source: Nutrients. 2020 Nov 4;12(11):3387. doi: 10.3390/nu12113387 (PMC7694211; doi:10.3390/nu12113387)
Supplement: Supplementary file 1 [file nutrients-12-03387-s001.docx]

Supplementary Table S1. Number of children and missing values.

|  | Dependent variable (outcome) | Number of parents or children included in mixed models with information at baseline or follow-up, or both | Number of parents or children participants at baseline | Number of parents or children participants at follow-up | Parents or children/ with missing values for PEL* | Number of missing values on variable who filled in the questionnaire | Children with missing values for age* |
| --- | --- | --- | --- | --- | --- | --- | --- |
| Parental role modelling | Screen time (min/day) ^a,c^ | 722 | 697 | 578 | 35 | 18 | 2 |
|  | Parental consumption of sugary everyday food and drinks (times/week)^a^ | 722 | 697 | 580 | 25 | 13 | 2 |
|  | Parental consumption of sugary treats (times/week) ^a^ | 722 | 697 | 579 | 23 | 12 | 1 |
|  | Parental fruit and vegetables consumption (times/week) ^a^ | 722 | 698 | 580 | 34 | 18 | 2 |
| Availability | Sugary everyday food and drinks at home (1-5) ^b^ | 642 | 620 | 519 | 29 | 16 | 0 |
|  | Sugary treats at home (1-5) ^b^ | 642 | 620 | 519 | 29 | 16 | 0 |
|  | Fruit and vegetables at home (1-5) ^b^ | 642 | 620 | 519 | 29 | 16 | 0 |
| Accessibility | Portable screens kept in-sight of the child ^b,c^ | 646 | 698 | 579 | 29 | 16 | 0 |
|  | Visits to outdoor PA places (1-5)^a^ | 722 | 591 | 498 | 35 | 18 | 2 |
|  | Sugary everyday food and drinks (1-2) ^b^ | 642 | 567 | 487 | 25 | 15 | 0 |
|  | Sugary treats (1-2) ^b^ | 633 | 583 | 495 | 25 | 15 | 0 |
|  | Fruit and vegetables (1-2) ^b^ | 625 | 619 | 517 | 25 | 15 | 0 |

PEL parental educational level, * only for models which are child based.

Supplementary Table S2a. Descriptive of outcomes- family environment by parental educational level (quantitative variables).

|  |  |  | Baseline | | | | Follow-up | | | |
| --- | --- | --- | --- | --- | --- | --- | --- | --- | --- | --- |
|  |  |  | Control | | Intervention | | Control | | Intervention | |
|  |  | PEL | n | mean ± SD* | n | mean ± SD* | n | mean ± SD* | n | mean ± SD* |
| Parental role modelling in presence of child | Screen time (min/day) | Low | 113 | 96.30 ± 66.39 | 109 | 87.09 ± 48.76 | 81 | 87.88 ± 63.67 | 81 | 80.24 ± 43.56 |
|  |  | Middle | 164 | 75.93 ± 44.01 | 143 | 71.43 ± 43.94 | 145 | 76.71 ± 55.98 | 116 | 70.44 ± 42.55 |
|  |  | High | 100 | 58.18 ± 42.37 | 56 | 70.64 ± 40.94 | 83 | 52.15 ± 39.70 | 49 | 63.24 ± 40.67 |
|  | Parental consumption of sugary everyday foods and drinks (times/week) | Low | 113 | 2.47 ± 2.54 | 108 | 2.42 ± 2.85 | 81 | 2.65 ± 2.96 | 82 | 2.24 ± 2.47 |
|  |  | Middle | 164 | 2.51 ± 2.40 | 143 | 1.88 ± 1.94 | 146 | 2.58 ± 2.46 | 116 | 2.13 ± 2.06 |
|  |  | High | 101 | 2.07 ± 1.97 | 56 | 2.62 ± 2.36 | 83 | 2.77 ± 3.00 | 49 | 3.04 ± 3.19 |
|  | Parental fruits and vegetables consumption (times/week) | Low | 113 | 6.00 ± 2.90 | 109 | 5.54 ± 2.86 | 81 | 5.93 ± 3.08 | 82 | 5.64 ± 2.80 |
|  |  | Middle | 164 | 6.34 ± 2.78 | 143 | 6.45 ± 2.60 | 146 | 6.34 ± 2.45 | 116 | 6.01 ± 2.87 |
|  |  | High | 101 | 6.54 ± 2.53 | 56 | 6.53 ± 2.41 | 83 | 6.74 ± 2.43 | 49 | 7.04 ± 2.49 |
| Availability | Sugary everyday food and drinks at home (1-5)^a^ | Low | 99 | 2.52 ± 0.69 | 96 | 2.58 ± 0.65 | 75 | 2.64 ± 0.71 | 71 | 2.52 ± 0.55 |
|  |  | Middle | 143 | 2.53 ± 0.61 | 127 | 2.55 ± 0.60 | 128 | 2.62 ± 0.61 | 104 | 2.52 ± 0.63 |
|  |  | High | 94 | 2.62 ± 0.64 | 51 | 2.65 ± 0.56 | 77 | 2.71 ± 0.67 | 45 | 2.96 ± 0.63 |
|  | Sugary treats at home (1-5)^a^ | Low | 99 | 2.67 ± 0.66 | 96 | 2.66 ± 0.65 | 75 | 2.73 ± 0.58 | 71 | 2.62 ± 0.57 |
|  |  | Middle | 143 | 2.88 ± 0.66 | 127 | 2.96 ± 0.61 | 128 | 2.87 ± 0.63 | 104 | 2.96 ± 0.63 |
|  |  | High | 94 | 2.98 ± 0.78 | 51 | 2.80 ± 0.55 | 77 | 3.04 ± 0.74 | 45 | 2.90 ± 0.62 |
|  | Fruits and vegetables at home (1-5)^a^ | Low | 99 | 4.02 ± 0.69 | 96 | 3.84 ± 0.72 | 75 | 4.06 ± 0.71 | 71 | 3.91 ± 0.75 |
|  |  | Middle | 143 | 4.15 ± 0.56 | 127 | 4.08 ± 0.62 | 128 | 4.16 ± 0.59 | 104 | 4.15 ± 0.64 |
|  |  | High | 94 | 4.16 ± 0.61 | 51 | 4.19 ± 0.56 | 77 | 4.31 ± 0.60 | 45 | 4.30 ± 0.57 |
| Accessibility | visits to outdoor PA places (1-5) | Low | 113 | 3.08 ± 0.72 | 109 | 3.10 ± 0.64 | 81 | 2.91 ± 0.80 | 81 | 2.96 ± 0.72 |
|  |  | Middle | 164 | 3.18 ± 0.62 | 143 | 3.20 ± 0.70 | 146 | 3.02 ± 0.68 | 116 | 3.00 ± 0.68 |
|  |  | High | 101 | 3.21 ± 0.62 | 56 | 3.20 ± 0.61 | 83 | 3.10 ± 0.73 | 49 | 3.14 ± 0.73 |
|  | Sugary food and drinks in-sight of the child (1-2)^b^ | Low | 92 | 1.02 ± 0.62 | 90 | 1.22 ± 0.51 | 71 | 1.13 ± 0.56 | 68 | 1.11 ± 0.56 |
|  |  | Middle | 139 | 1.08 ± 0.52 | 122 | 0.99 ± 0.54 | 121 | 1.04 ± 0.46 | 102 | 1.02 ± 0.53 |
|  |  | High | 90 | 0.93 ± 0.54 | 48 | 1.00 ± 0.59 | 73 | 1.09 ± 0.50 | 45 | 0.92 ± 0.64 |
|  | Sugary treats in-sight of the child (1-2)^b^ | Low | 88 | 1.26 ± 0.53 | 86 | 1.22 ± 0.52 | 68 | 1.32 ± 0.49 | 65 | 1.25 ± 0.50 |
|  |  | Middle | 138 | 1.23 ± 0.42 | 113 | 1.22 ± 0.41 | 120 | 1.19 ± 0.44 | 99 | 1.23 ± 0.41 |
|  |  | High | 87 | 1.12 ± 0.38 | 46 | 1.13 ± 0.39 | 74 | 1.24 ± 0.45 | 43 | 1.17 ± 0.47 |
|  | Fruits and vegetables in-sight of the child (1-2)^b^ | Low | 87 | 1.83 ± 0.29 | 93 | 1.87 ± 0.25 | 70 | 1.89 ± 0.23 | 66 | 1.90 ± 0.22 |
|  |  | Middle | 139 | 1.88 ± 0.24 | 120 | 1.83 ± 0.29 | 124 | 1.83 ± 0.27 | 99 | 1.84 ± 0.29 |
|  |  | High | 88 | 1.85 ± 0.30 | 48 | 1.86 ± 0.29 | 74 | 1.78 ± 0.31 | 45 | 1.88 ± 0.26 |

SD* Standard deviation

^a^ The questions on food availability had five answer categories; never, rarely, sometimes, often, and always, which yielded 1,2,3,4, and 5 points respectively. The points from of the five questions were summed up by participant, and the mean was used in analysis.

^b^ The questions on accessibility of food had two answer categories; no or yes, which yielded 1 or 2 points respectively. The points were summed up, and a mean of the three separate variables by participant was used in the analysis

Supplementary Table S2b. Descriptive of the outcomes- family environment by parental educational level (categorical variables).

|  |  |  | Baseline | | | | Follow-up | | | |
| --- | --- | --- | --- | --- | --- | --- | --- | --- | --- | --- |
|  |  |  | Control | | Intervention | | Control | | Intervention | |
|  | PEL |  | Number | percentage (%) | Number | percentage (%) | Number | percentage (%) | Number | percentage (%) |
| Parental role modelling of sugary treats | Low | not at all | 35 | 31 | 39 | 36 | 23 | 28 | 29 | 35 |
|  |  | 1-2 times | 70 | 62 | 59 | 55 | 48 | 59 | 45 | 55 |
|  |  | 3-4 times | 4 | 3 | 7 | 6 | 7 | 9 | 6 | 7 |
|  |  | 5-6 times | 1 | 1 | 3 | 3 | 2 | 2 | 0 | 0 |
|  |  | once everyday | 1 | 1 | 0 | 0 | 1 | 1 | 2 | 2 |
|  |  | more than once a day | 2 | 2 | 0 | 0 | 0 | 0 | 0 | 0 |
|  | Middle | not at all | 44 | 27 | 34 | 24 | 30 | 20 | 18 | 16 |
|  |  | 1-2 times | 87 | 53 | 93 | 65 | 83 | 57 | 72 | 63 |
|  |  | 3-4 times | 29 | 18 | 12 | 8 | 26 | 18 | 20 | 17 |
|  |  | 5-6 times | 4 | 2 | 2 | 1 | 5 | 3 | 1 | 1 |
|  |  | once everyday | 0 | 0 | 1 | 1 | 1 | 1 | 4 | 3 |
|  |  | more than once a day | 0 | 0 | 1 | 1 | 0 | 0 | 0 | 0 |
|  | High | not at all | 22 | 22 | 20 | 20 | 18 | 22 | 9 | 18 |
|  |  | 1-2 times | 61 | 60 | 70 | 70 | 42 | 51 | 28 | 57 |
|  |  | 3-4 times | 16 | 16 | 5 | 5 | 16 | 19 | 8 | 16 |
|  |  | 5-6 times | 2 | 2 | 0 | 0 | 5 | 6 | 1 | 2 |
|  |  | once everyday | 0 | 0 | 5 | 5 | 2 | 2 | 3 | 6 |
|  |  | more than once a day | 0 | 0 | 0 | 0 | 0 | 0 | 0 | 0 |
| Accessibility of screens | Low | Strongly agree | 44 | 45 | 47 | 49 | 32 | 43 | 21 | 30 |
|  |  | Agree | 33 | 34 | 31 | 32 | 26 | 35 | 30 | 42 |
|  |  | Neither agree nor disagree | 8 | 8 | 7 | 7 | 7 | 9 | 7 | 10 |
|  |  | Disagree | 8 | 8 | 5 | 5 | 6 | 8 | 6 | 8 |
|  |  | Strongly disagree | 5 | 5 | 6 | 6 | 3 | 4 | 7 | 10 |
|  | Middle | Strongly agree | 68 | 48 | 58 | 46 | 58 | 45 | 45 | 44 |
|  |  | Agree | 48 | 34 | 46 | 36 | 46 | 36 | 40 | 39 |
|  |  | Neither agree nor disagree | 9 | 6 | 9 | 7 | 7 | 5 | 6 | 6 |
|  |  | Disagree | 6 | 4 | 7 | 5 | 9 | 7 | 7 | 7 |
|  |  | Strongly disagree | 12 | 8 | 7 | 5 | 8 | 6 | 5 | 5 |
|  | High | Strongly agree | 44 | 47 | 22 | 43 | 39 | 51 | 25 | 56 |
|  |  | Agree | 38 | 38 | 26 | 51 | 28 | 36 | 18 | 40 |
|  |  | Neither agree nor disagree | 3 | 3 | 1 | 2 | 3 | 4 | 1 | 2 |
|  |  | Disagree | 9 | 9 | 1 | 2 | 6 | 8 | 1 | 2 |
|  |  | Strongly disagree | 2 | 2 | 1 | 2 | 1 | 1 | 0 | 0 |
